# Identifying hotspots for ecosystem restoration across heterogeneous tropical savannah-dominated regions

**DOI:** 10.1098/rstb.2021.0075

**Published:** 2023-01-02

**Authors:** Kennedy Lewis, Fernanda de V. Barros, Peter W. Moonlight, Timothy C. Hill, Rafael S. Oliveira, Isabel B. Schmidt, Alexandre B. Sampaio, R. Toby Pennington, Lucy Rowland

**Affiliations:** ^1^ College of Life and Environmental Sciences, University of Exeter, Exeter, Devon EX4 4QE, UK; ^2^ Tropical Diversity Section, Royal Botanic Gardens Edinburgh, Edinburgh EH3 5LR, UK; ^3^ Department of Plant Biology, Institute of Biology, University of Campinas, Campinas, CEP 13083-970, Brazil; ^4^ Department of Ecology, University of Brasília, Brasília, CEP 70.910-900, Brazil; ^5^ Centro Nacional de Avaliação da Biodiversidade e de Pesquisa e Conservação do Cerrado CBC, Instituto Chico Mendes de Conservação da Biodiversidade – ICMBio, University of Brasília, Brasília, CEP 70.670-350, Brazil

**Keywords:** restoration, savannah regions, Brazilian Cerrado, biodiversity, biomass, landscape connectivity

## Abstract

There is high potential for ecosystem restoration across tropical savannah-dominated regions, but the benefits that could be gained from this restoration are rarely assessed. This study focuses on the Brazilian Cerrado, a highly species-rich savannah-dominated region, as an exemplar to review potential restoration benefits using three metrics: net biomass gains, plant species richness and ability to connect restored and native vegetation. Localized estimates of the most appropriate restoration vegetation type (grassland, savannah, woodland/forest) for pasturelands are produced. Carbon sequestration potential is significant for savannah and woodland/forest restoration in the seasonally dry tropics (net biomass gains of 58.2 ± 37.7 and 130.0 ± 69.4 Mg ha^−1^). Modelled restoration species richness gains were highest in the central and south-east of the Cerrado for savannahs and grasslands, and in the west and north-west for woodlands/forests. The potential to initiate restoration projects across the whole of the Cerrado is high and four hotspot areas are identified. We demonstrate that landscape restoration across all vegetation types within heterogeneous tropical savannah-dominated regions can maximize biodiversity and carbon gains. However, conservation of existing vegetation is essential to minimizing the cost and improving the chances of restoration success.

This article is part of the theme issue ‘Understanding forest landscape restoration: reinforcing scientific foundations for the UN Decade on Ecosystem Restoration’.

## 1. Introduction

The United Nations Decade on Ecosystem Restoration (2021–2030) aims to ‘prevent, halt and reverse the degradation’ of a wide range of ecosystems [1]. Restoration is an important tool for preserving remaining biodiversity and for maintaining ecosystem services [2]. Restoration projects may also function as nature-based solutions to climate change and underpin emission reduction targets for multiple stakeholders at various scales [3]. In order to prevent further extinctions and escalating climate disaster, widespread ecosystem restoration, alongside conservation, must be undertaken rapidly [4,5]. Strategic and effective approaches to restoration, which use landscape-scale restoration planning and prioritization to optimize restoration benefits, will therefore be essential [2,6].

The tropics have been at the centre of recent ecosystem degradation due to the expansion of agriculture and pasturelands, and as a result, the area of restorable land in tropical regions is high [7,8]. Around half of the global tropical land area comprises seasonally dry biomes, where vegetation experiences intense seasonal water deficit [9]. Seasonally dry, tropical savannah-dominated regions cover approximately 20% of the global land surface and are characterized by a mixture of grassy and woody vegetation, from open grassland to closed canopy forest communities [10]. This heterogeneity in vegetation type is driven by variable fire regimes, complex geology, herbivory and strong gradients in soil nutrient and water availability [11], producing a mosaic-like landscape of different vegetation types that coexist even at small spatial scales [12–14]. As a result, savannah-dominated regions often have a high local plant species diversity [15,16] and a high turnover in species composition [17], resulting in high overall species richness. The contribution of seasonally dry biomes to the global carbon and water balance is increasingly gaining attention [18,19] and they are often relied upon by local communities for the provision of fuelwood and other goods [20]. Despite their value, seasonally dry tropical biomes, including savannah-dominated regions, have suffered extensive historical and accelerating degradation and land-use change when compared to other tropical biomes [21,22]. However, relative to humid tropical forests, little attention has been given to their restoration, or to improving our understanding of how to restore seasonally dry tropical biomes [9,23–26]. The potential benefits of restoration in these regions remain relatively unexplored and unquantified [24,25], meaning the rate of implementation of restoration has also been comparatively slow.

Restoration of vegetation across savannah-dominated regions is challenging because of the mosaic nature of their landscapes. First, the heterogeneity of vegetation types and their specificity to soil and fire regime conditions means that a single restoration approach is not appropriate across wide spatial scales [26,27]. Often the previous vegetation cover (if known) and neighbouring remaining native vegetation are likely to be good indicators of the most appropriate vegetation types to restore [23,27]. Remaining native vegetation is also a good source for propagules of species appropriate for use in restoration when harvested sustainably [28]. Second, natural regeneration potential is often low after intense soil disturbance [29], necessitating active restoration techniques, which must be resilient to fire [30], drought events [31] and the high risk of invasion from competitive exotic planted pasture grasses [27,32]. Furthermore, the impacts of rising atmospheric CO_2_ concentrations on the climate are expected to lead to increased surface temperatures (ST) with regional variation in the direction of precipitation change trends across the seasonally dry tropics [33]. Restoration projects must be resilient to these changes, and potential increases in aridity [34], to prevent climate-related restoration failure [31,35]. Overcoming these risks to restoration success requires increased investment into understanding restoration techniques [36], something that is more likely to be achieved if the trade-off between restoration benefits and potential risks can be evaluated at scale, as has been done in other tropical regions [7].

Successful and appropriate restoration is dependent on the consideration of many factors and requires extensive planning [37,38]. Legislation often determines the area, type and location of land that stakeholder groups are required to conserve and restore [3,36,39,40]. In addition to this, restoration should be carried out in such a way that the necessary rates of food and resource production are maintained and the displacement of production and leakage of degradation elsewhere are minimized [38]. Initiating and implementing restoration projects will likely require the mobilization and engagement of a diverse group of local, private and international stakeholders [3,40]. The likelihood of investment in restoration hinges on the cost of restoration and the risk of restoration failure [36]. Restoration hotspots—areas with the greatest payback in terms of restoration benefits and where it might be easier, and therefore potentially cheaper, to successfully implement restoration—will be ideal locations to focus initial restoration efforts [2,6]. Carbon sequestered through restoration is marketable and therefore important for attracting investment in restoration projects [41]. Despite this, the potential species richness gained through restoration should be given equal consideration [4,38]. This balance is of particular importance across savannah vegetation, where a trade-off between vegetation biomass and species richness has been observed in multiple studies, due to increases in woody vegetation density causing a loss of the shade-intolerant herbaceous stratum [42]. Few studies have attempted to identify restoration hotspots for mosaic landscapes across savannah-dominated regions.

**Figure 1.**
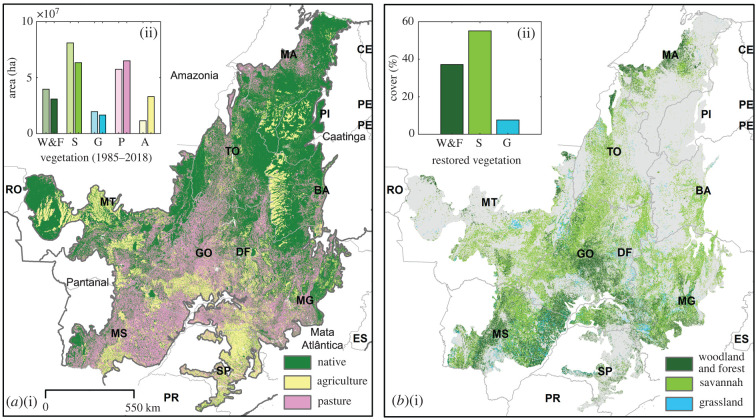
Cerrado landcover and potential restoration vegetation type. (*a*,i) 2018 Cerrado vegetation cover, adapted from MapBiomas collection 5 at a 100 m resolution. (*a*,ii) Total area (ha) of the Cerrado covered by woodland & forest (W&F), savannah (S), grassland (G), pastureland (P) and agriculture (including forest plantations) (A) in 1985 (left bar) and 2018 (right bar). Boundaries of the major Brazilian boundaries boarding the Cerrado are indicated (Amazonia, Mata Atlântica, Caatinga and Pantanal). See electronic supplementary material, figure S2 for full biome extents. (*b*,i) Potential restoration vegetation cover type (grassland, savannah and W&F) across all areas classified as pasturelands in 2018, based on previous native vegetation cover and proximity to remaining native vegetation. (*b*,ii) Target proportion of each restored vegetation type as a percentage of the potential restoration area. Brazilian states indicated included Bahia (BA), Ceará (CE), Distrito Federal (DF), Espírito Santo (ES), Goiás (GO), Maranhão (MA), Minas Gerais (MG), Mato Grosso do Sul (MS), Mato Grosso (MT), Pernambuco (PE), Piauí (PI), Paraná (PR), Rondônia (RO), São Paulo (SP) and Tocantins (TO).

The Brazilian Cerrado is a tropical savannah-dominated region covering ∼2 million km^2^ [17] and has undergone extensive land-use change over the past six decades (figure 1*a*, [12,43]). Despite the dominance of savannah formations, vegetation across the Cerrado is highly heterogeneous, including various types of grassland, savannah, woodland and forest [10] (electronic supplementary material, table S1). It is the world's most species-rich savannah region with total plant species equalling that of the Brazilian Amazon and 36.7% of its species diversity endemic [15,16]. In addition, the vegetation also stores considerable amounts of carbon, particularly in belowground biomass [44] and plays an important role in regulating and recharging water sources both within and outside of the Cerrado region [45]. Consequently, the need to conserve remaining vegetation and the potential to undertake ecosystem restoration are both high [46]. Across the Cerrado, 2.1 Mha of degraded land has been targeted for restoration by 2030 [47]. The restoration of Cerrado vegetation types, in addition to restoration across other Brazilian biomes, is critical if Brazil's emission reduction targets under the UNFCCC Paris Agreement are to be met [48], making it an excellent case study for understanding how to restore these complex savannah-dominated systems.

Here, using the Brazilian Cerrado as the focus of our analysis, we propose a methodology for identifying hotspots for restoration in complex mosaic landscapes across tropical savannah-dominated regions to facilitate large-scale restoration planning and highlight restoration benefits. Our first objective is to produce spatially explicit estimates of the most appropriate vegetation type to restore in planted pasturelands across the Cerrado region. Next, an assessment of how the potential carbon sequestration and plant biodiversity benefits of restoration may covary spatially is carried out. Then, as an indicator of the likelihood of restoration success, possible connectivity with existing native vegetation is considered (because this increases natural regeneration potential and decreases exotic invasion risk). These metrics are then combined to identify restoration hotspots across the Cerrado region. Finally, how regional legislation regulating land use and management might affect the feasibility of restoring these areas, and how predicted climatic changes may impact these hotspots, is assessed.

## 2. Methods

Existing maps of current and historic landcover were analysed to assess the most appropriate potential restoration vegetation type (§2a). Three benefit and success metrics from restoration were then assessed: §2b(i) potential plant species diversity gains, derived from species distribution models (SDMs); §2b(ii) net total biomass change from restoration, derived from plot aboveground biomass (AGB) and belowground biomass (BGB) inventories and a mapped AGB product; and §2b(iii) potential connectivity with remaining native vegetation in the surrounding area. These three metrics were used to identify hotspots for restoration in the Cerrado region, for each vegetation type (§2c), which are then assessed with reference to Brazil's Forest Code (§2d). Downscaled global climate model (GCM) outputs were then used to assess potential temperature changes across the region (§2b(iv)). A full flowchart of the methods employed in this study is presented in electronic supplementary material, figure S1.

### (a) Potential restoration vegetation type

Three broad vegetation classes; grassland, savannah and woodland and forest (W&F), are considered suitable for restoration across the Cerrado region (electronic supplementary material, figure S2). Across the Cerrado, localized estimates of the appropriate vegetation cover for land that could potentially be restored were assessed using the MapBiomas (collection 5) landcover maps (electronic supplementary material, figure S3, [12,22]) within the Google Earth Engine platform [49]. All planted pastures in 2018 were considered potential areas for restoration. A large proportion of Cerrado pasturelands are now degraded (39%, [50]), abandoned or underutilized, meaning an area of up to approximately 6.4 Mha could be restored to native vegetation by 2050, while maintaining projected increases in food production [46]. Although it is not feasible to restore all Cerrado pasturelands to native vegetation [46], and land opportunity costs will vary across the Cerrado [50], we outline potential restoration benefits across all planted pastures.

Pre-land-use change vegetation type and remaining neighbouring native vegetation cover are good indicators of local abiotic conditions [11,27]. In pasturelands where the native vegetation cover type (grassland, savannah or W&F) in 1985 was known, the per hectare restoration type was assigned to match this historical vegetation cover. If the previous (1985) native vegetation cover was unknown, the restoration cover type was assigned based on proximity to remaining 2018 and known 1985 native vegetation. See electronic supplementary material, Section 1*a* for extended methodologies.

### (b) Restoration benefit and success metrics

#### (i) Plant species richness

Potential species richness for each restoration vegetation type across the Cerrado region was estimated using SDMs. All Brazilian specimen records from the CRIA Species Link [51] and the Reflora Virtual herbarium [52] databases were retrieved; see electronic supplementary material, Section 1*b*. Data were cleaned in six stages to remove records with georeferencing errors (see electronic supplementary material, appendix S2 of Moonlight *et al*. [13]). Environmental bias in occurrence data was minimized by retaining only a single occurrence record within a 10 km radius for each species, following Kramer-Schadt *et al*. [53]. Species with fewer than five records were excluded, and the final dataset included 391 993 records for 12 583 species.

Climatic and edaphic predictors were used at a 0.05° resolution (approx. 5.5 km at the equator). Climate data were calculated from CHELSA [54] monthly gridded climatologies, including annual MI (the ratio of annual rainfall to equilibrium evapotranspiration) using the Priestly-Taylor equation [55]; minimum monthly MI; dry season length (number of months with an MI < 1); minimum temperature of the coldest month (bio6) and number of days above 25°C. Edaphic variables were obtained from the SoilGrids 250 m database [56]. Fifty-five edaphic variables were converted into five principal components analysis (PCA) axes that explained more than 80% of the variation, therefore maximizing explanatory power while reducing the risk of model overfitting (appendix S2 in [13]); see electronic supplementary material, section 1*b*.

SDMs were run under MaxEnt v. 3.3.3 in the ‘dismo’ package in R [57] under the default MaxEnt settings, with all feature classes allowed and with 5-fold cross validation. SDMs were trained with 10 000 background points sampled using an Epanechnikov kernel calculated from all Angiosperm distribution data for Brazil (following Weigand & Moloney [58]). SDM performance was evaluated using the continuous Boyce index (CBI, where a model with a CBI > 0 is considered better than random [59,60]). Here, models with a CBI < 0.25 were discounted from our further analyses, leaving 8791 species.

Many Cerrado region species are found only in either grassland, savannah or W&F stands [14], so a separate potential species richness layer was calculated for each vegetation type. Modelled species were assigned to one or more vegetation types based upon the vegetation classification in Flora do Brasil [16] and their corresponding MapBiomas landcover class (see electronic supplementary material, table S1). In recognition of the fact that savannah vegetation with high woody cover (cerradão) is classified as forest in MapBiomas [12], 1138 woody species from the Flora do Brasil vegetation type ‘cerrado *sensu lato*’ were also classified under W&F. Across Brazil, this resulted in 6233 W&F, 2525 grassland and 3930 savannah species. Models were summed from each vegetation type across the Cerrado region to provide a potential species richness map for each restoration type.

#### (ii) Net vegetation biomass change

The estimated net total biomass (AGB + BGB) gained through restoration was calculated for each potential restoration hectare, based on the assigned restoration vegetation type. Localized per vegetation type AGB stock estimates (informed by the MapBiomas landcover) for 2018 were extracted from the ESA CCI_Biomass AGB product (Mg ha^−1^, 0.1 km resolution) [61]. These maps of the remaining native vegetation AGB stocks were then used to produce localized estimates of the potential AGB stock of restored vegetation for all three vegetation types. Finally, empirical inventory data were used to validate and adjust the localized potential AGB stock estimates and maintain a realistic distribution of AGB stocks and trends (see electronic supplementary material, §1*c*, figure S4).

A literature review of AGB and BGB plot inventories within the Cerrado was carried out (W&F : *n* = 21, savannah: *n* = 86, grassland: *n* = 29, electronic supplementary material, table S2). Where both AGB and BGB were measured at inventory plot sites (*n* = 39), root : shoot ratios (R:S) were calculated. A mean R:S for each vegetation type was applied to the adjusted localized restoration AGB to obtain the potential mature total biomass stock for each restoration hectare. An estimated total biomass stock for plated pastures was used to calculate the net biomass change of restoration (electronic supplementary material, §1*c*). Potential changes in soil organic carbon stocks (SOC) were not included in this analysis, as observations of the change in SOC post restoration were not available in the literature.

#### (iii) Connectivity and surrounding vegetation matrix

The composition of the vegetation matrix surrounding a potential restoration hectare is used as a proxy for optimizing some restoration benefits and minimizing some potential risks. Total native vegetation cover surrounding a potential restoration hectare is calculated using a 1.21 km^2^ moving window (approx. 0.5 km radius) and is expressed as a percentage of the window area. A higher proportion of native vegetation cover in the area surrounding a potential restoration site increases potential harvestable propagule availability and the likelihood of some natural regeneration occurring (for vegetation that does not rely on resprouting of underground structures) [27,28,62]. It also reduces the likelihood of invasion from exotic planted pasture species leading to restoration failure [32,63]. The higher the percentage of native vegetation, the greater the likelihood that native vegetation fragments might be connected by restoration [64]. To assess the effect of varying the size of the moving window on the distribution of hotspot areas, a sensitivity analysis was undertaken using alternative window sizes between 0.21 and 9.61 km^2^ (see electronic supplementary material, figure S5). The resultant hotspot scores, and the location of priority areas, were not very sensitive to the size of the moving window, suggesting our connectivity metric was robust to changes in spatial scaling.

#### (iv) Climate risk

Predicted increases in maximum surface temperatures (ST_max_) were used as a proxy for climate change risk. Rising STs may increase the risk of long-term restoration failure [31,35,40], suggesting areas with high anticipated increases in ST should be avoided for restoration. Conversely, these areas may also be the most in need of restoration, as native vegetation can help to increase water infiltration and prevent temperature rises from leading to desertification [7,40]. Given these potentially opposing effects, climate risk is not included in the hotspot calculations and is analysed separately.

To assess variation in predicted ST_max_, climate risk maps are produced using 4 downscaled CMIP5 global climate model outputs (ACCESS1-3, MIROC5, CESM1-BGC, CMCC-CM, [65]). Predicted ST_max_ under the IPCC's Representative Climate Pathway 8.5 (RCP 8.5) was acquired at a 5 km resolution and monthly time-step for 2006–2016 (initial) and 2090–2100 (end). The mean absolute change in ST_max_ was calculated between these two periods [66]. Multi-model uncertainty (variability in temperature increase between models) was expressed as the standard error of the mean.

### (c) Hotspot analysis

Ecosystem restoration benefit and success metrics; plant species richness, potential net total biomass gain and native vegetation connectivity were all equally weighted and considered independently for each vegetation class. Each potential benefit or success metric map, for each vegetation class, was box-cox transformed to normalize the distribution of the dataset while preserving order. All transformed datasets were then scaled from 0 to 1. A per hectare mean score, considering all metrics, was then scaled from 0 to 1 to produce each hotspot map. When assessing grassland restoration hotspots net total biomass change was not included, as changes in biomass stocks following planted pastureland conversion to native grassland species are assumed to be negligible or may even result in a biomass loss. A combined Cerrado-wide restoration hotspot map, assuming all pasturelands are restored to their most appropriate vegetation type, was also produced. To aid visualization, mean per hectare scores are averaged to a 5.5 km resolution across all potential pasture pixels in the final restoration hotspot maps.

### (d) Land use legislation

The resulting map of restoration hotspot areas was assessed with reference to Brazil's Forest Code. The Forest Code legally defines the proportion of a given private rural property (outside of legally protected areas) where native vegetation must be conserved, although some supervised sustainable management is allowed [39]. Microwatershed scale maps of the estimated Forest Code balance, where areas in vegetation debt must be restored and areas in surplus may legally undergo removal of native vegetation, were obtained from Soares-Filho *et al.* [43]. This analysis allows an assessment of the likelihood of native vegetation clearance or restoration necessitated by legislation in hotspot locations across the region at a coarse spatial scale.

## 3. Results

### (a) Cerrado landcover change and potential restoration vegetation type

Across the Cerrado region, in 2018, approximately 53% of the vegetated area comprised native vegetation: grassland (8%), savannah (31%) and W&F (14%) (figure 1*a*). Between 1985 and 2018 the area of native vegetation cover reduced by 20.9% (figure 1*a*). In 1985 a large proportion of the Cerrado region was already being used as pastureland (27%). As a result, the historical native vegetation type can only be identified for 38% of areas classified as pastures in 2018, of which 3, 24 and 12% were previously grasslands, savannahs and W&F respectively. When attempting to assess the most appropriate local vegetation type for restoration, savannah was the most commonly assigned class (55%; figure 1*b*), followed by W&F (37%), which was most common in the southern Cerrado region and the Cerrado-Amazonia and Cerrado-Mata Atlântica transition zones (figure 1*b*). Areas where restoration to grasslands is likely make up 8% of total pasture area, mostly across the southern and central Cerrado.

### (b) Potential restoration benefits and success metrics

#### (i) Plant species richness

**Figure 2.**
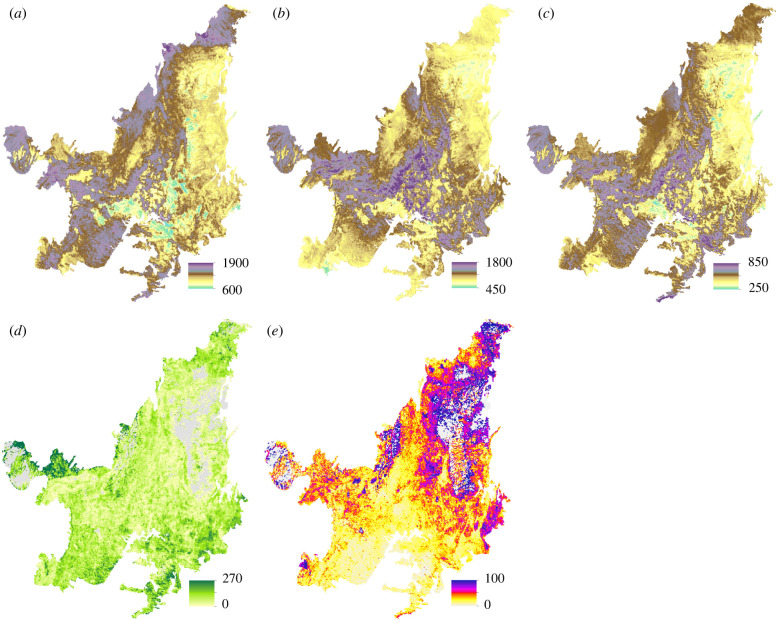
Potential restoration gain and success metrics. Modelled woodland & forest (*a*), savannah (*b*) and grassland (*c*) vegetation species richness (no. species). Colourbar limits vary between panels to facilitate the assessment of spatial variation in modelled species richness for each vegetation type. To aid inter-vegetation type comparison, panels (*a*–*c*) are presented with a standardized colourbar in electronic supplementary material, figure S6. (*d*) Estimated net total biomass (AGB + BGB) gain following restoration (Mg ha^−1^, mature restored vegetation), based on the restoration type in figure 1*b* (electronic supplementary material, figure S7). (*e*) Proportion of native vegetation within the matrix surrounding a potential restoration hectare (native vegetation area within a 1.21 km^2^ window (%)). Maps in panels (*d*) and (*e*) have been averaged to a 5.5 km resolution for display purposes.

The probability of a species occurring within the Cerrado was greater than 75% for 7950 of the species modelled (figure 2*a–c*). Modelled potential plant species richness was high for W&F vegetation, with a maximum predicted diversity of 1878 species within a 5.5 km grid-cell and a mean modelled diversity of 1237 species (±210 s.d.) for grid cells across the Cerrado (figure 2*a*). However, of the species included in the W&F species richness maps, 18% were woody savannah species (‘cerrado *sensu* lato’, electronic supplementary material, table S1). Modelled W&F species richness was highest in the western half of the Cerrado, particularly in the Cerrado–Amazonia transitional zone (figure 2*a*). Similarly, the modelled grid-cell diversity of savannah vegetation was also high, with a maximum and mean of 1773 and 1028 species (±222 s.d.) across the Cerrado region, respectively. By contrast to W&F, modelled savannah species richness was highest across the central belt of the Cerrado in Goiás, Minas Gerais and Mato Grosso (figure 2*b*). Regionally, 1351 species occurred in both the modelled savannah and grassland vegetation species richness maps (54% of grassland species; 34% of savannah species). Grassland vegetation had a mean modelled grid-cell diversity of 491 (±91 s.d.) and maximum of 827 species, with a high species richness across the central belt and the northern and southern extremes of the Cerrado (figure 2*c*).

#### (ii) Net vegetation biomass gain

**Figure 3.**
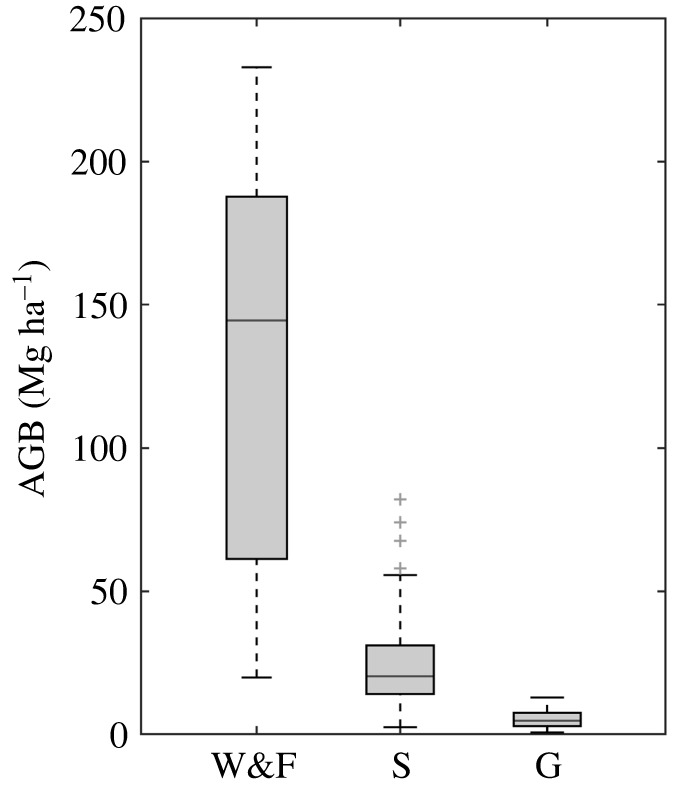
Woodland and forest (W&F, *n* = 21), savannah (S, *n* = 86) and grassland (G, *n* = 29) aboveground biomass stocks as reported from empirical plot inventory studies across the Cerrado.

Across the collated plot inventories, the mean (and s.d.) AGB stocks for grasslands, savannahs and W&F vegetation were 5.3 (±3.2), 24.3 (±16.2) and 127.6 (±68.9) Mg ha^−1^, respectively (figure 3). As expected, the R:S was markedly different across the vegetation types and was highest for grassland vegetation with a mean (and s.d.) R:S of 2.3 ± 2.2 (*n* = 14) and savannahs with a R:S of 1.8 ± 0.7 (*n* = 21). W&F had a lower R:S of 0.3 ± 0.2, with fewer studies reporting BGB data (*n* = 4). The mean (and s.d.) potential total biomass gain of restoration across the Cerrado was 58.2 ± 37.7 Mg ha^−1^ for savannahs and 130.0 ± 69.4 Mg ha^−1^ for W&F (figure 2*d*). The spatial distribution of potentially high total biomass gains is similar for both the savannah and W&F restoration types, with high estimated biomass gains in areas where the Cerrado transitions into forest-dominated regions in Mato Grosso (Amazonia), Minas Gerais and São Paulo (Mata Atlântica) and in Maranhão (Caatinga and Amazonia) (electronic supplementary material, figure S7).

#### (iii) Connectivity and surrounding vegetation matrix

At a 1 ha resolution, approximately 8% of pasturelands have no native vegetation within a 1.21 km^2^ window within the Cerrado. On average, only 24% of the matrix surrounding a restoration pixel contains native vegetation (within a 1.21 km^2^ area). The mean proportion of native vegetation cover surrounding a pasture hectare is lowest in Mato Grosso do Sul (15%), Goiás (23%) and São Paulo (15%), corresponding to high pastureland and agricultural coverage (figures 1*a*(i) and 2*e*). Native vegetation connectivity is greatest (37–60%) across the states in the north and northeast, along the Cerrado–Caatinga transition (figure 2*e*). However, by definition, areas with a higher connectivity have less pastureland area that might be targeted for restoration (figure 1*b*(i)).

### (c) Woodland and forest, savannah and grassland restoration hotspots

**Figure 4.**
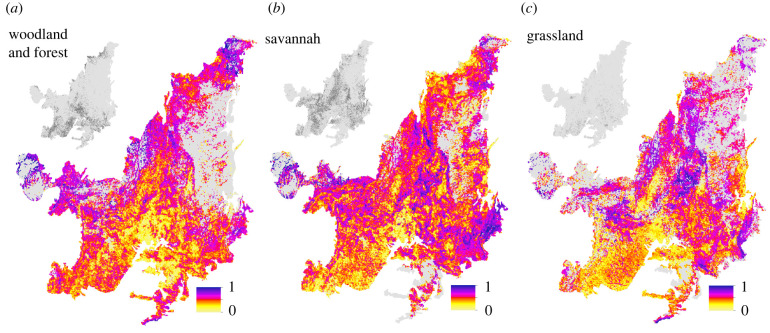
Hotspot analysis for each restoration vegetation type. Woodland & forest (*a*), savannah (*b*) and grassland (*c*) total potential restoration benefit and success scores. Restoration scores (0–1) accounting for potential plant species richness, net total biomass gain (mature vegetation, W&F and savannah restoration only) and proportion of native vegetation in the local vegetation matrix. Score maps are averaged to a 5.5 km resolution for display purposes (and are not representative of local pastureland area). The appropriate vegetation type assigned considering all pasturelands is indicated in the top left of each panel (dark grey).

When all potential restoration benefits and success metrics (species richness, total biomass gain and connectivity, all considered of equal importance) are normalized and combined, hotspot areas vary for grassland, savannah and W&F vegetation, but medium- (greater than 0.5), high- (greater than 0.7) and very high- (greater than 0.8) scoring areas are distributed across the Cerrado region for all vegetation classes (figure 4 and electronic supplementary material, figure S8). Our analysis also demonstrates that for savannah and W&F types across the Cerrado region, high-scoring areas for plant species richness rarely correspond to high-scoring areas for net biomass gain (electronic supplementary material, figure S9).

Northern Goiás and Tocantins have a high density of very high-scoring areas for both savannah and grassland restoration (figure 4*b,c*). The northern portion of the Cerrado region, in close proximity to both Amazonia and the Caatinga, is high-scoring for W&F and savannah restoration. The Cerrado–Mata Atlântica transition in the eastern state of Minas Gerais also has a high density of high- to very high-scoring areas for all restoration types, particularly savannahs (figure 4). Central and western Mato Grosso (in the far west of the Cerrado) and the Mato Grosso–Rondônia border also score highly and very highly for all restoration types (figure 4). When assessed at the microwatershed scale, several of these hotspot locations are however in areas with a surplus of native vegetation under the Forest Code as calculated by Soares-Filho *et al*. [43] (electronic supplementary material, figure S10*a*–*c*), meaning that restoration is not legally required, and removal of remaining native vegetation may be permitted.

### (d) Restoration hotspots and climate risk

**Figure 5.**
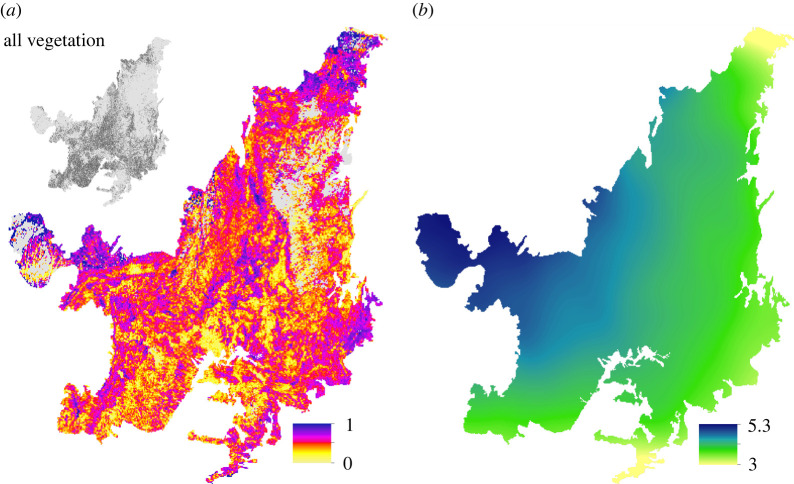
Hotspot analysis (total potential restoration benefit and success score) and climate risk across all restoration vegetation types. (*a*) Restoration score (0–1) accounting for potential plant species richness, net total biomass gain (W&F and savannah restoration only) and proportion of native vegetation in the local vegetation matrix. Scores are averaged to a 5.5 km resolution for display purposes and will therefore reflect the restoration type with the largest potential area coverage within the corresponding 30.25 km^2^ area (figure 1*b*). (*b*) Predicted absolute increase in ST_max_ (°C) between 2006 and 2100 under the IPCC Representative Concentration Pathway (RCP) 8.5.

When all potential restoration vegetation types are considered, restoration hotspots broadly mirror those identified for each formation type as previously outlined (figures 4 and 5*a*). Critically, four areas appear to have high concentrations of high-scoring land. These are located in the central (northern Goiás and Tocantins), the northern (Maranhão), eastern (Minas Gerais) and far west of the Cerrado region (Mato Grosso and Rondônia). However, the predicted climate change risk varies substantially across these regions (figure 5*b*). Initial mean ST_max_ ranged from 21.3 to 33.1°C across the Cerrado region (electronic supplementary material, figure S11*a*). Between 2006 and 2100 predicted absolute increases in ST_max_ range from 3.11 to 5.29°C under RCP 8.5. The mean (and s.d.) multi-model uncertainty across the region were 0.55 ± 0.11°C (electronic supplementary material, figure S11*b*). Extremes within the range of predicted absolute ST_max_ increases across the Cerrado often correspond with restoration hotspots; for example, in the far west, in Mato Grosso where the predicted increase in temperature is high, and in the far north, in Maranhão where increases are lowest (figure 5*b*).

## 4. Discussion

After producing fine-scale estimates of the most appropriate restoration type across heterogeneous landscapes in our large savannah-dominated study region, we find significant potential gains in species richness and carbon stocks through restoration of vegetation across all areas. Four key locations across the Brazilian Cerrado that have a high concentration of areas with high combined restoration benefits are identified. The central Cerrado was an important hotspot area for savannah and grassland restoration. The other three hotspot locations were in transitional areas between biomes, where cross-biome species mixing may increase species diversity and total biomass stocks were often high. These hotspot areas have a high species diversity and potential carbon sequestration gain from restoration but are also all located in areas where native vegetation was less fragmented, increasing potential connectivity with existing native vegetation. This will likely increase the success and reduce the cost of restoration. By contrast, areas with the greatest proportion of land potentially available, and where legislation often enforces the necessity of restoration, were not highlighted as hotspots. Our results emphasize the diversity of restoration approaches that are likely to be required across the seasonally dry tropics and the potential dangers associated with categorizing these regions as comprising a single vegetation type (e.g. savannah) within land use policy and restoration science [67].

Planning ecosystem restoration across regions made up of heterogeneous vegetation mosaics initially requires careful consideration of the most appropriate restoration vegetation type [26,27]. However, broad vegetation classes (e.g. grassland, savannah and W&F) still cannot fully reflect the extensive variation in vegetation characteristics and functioning observed in the distinct physiognomies found within each category, which should be accounted for in future restoration planning attempts (electronic supplementary material, table S1). In addition, determining a vegetation reference state in some seasonally dry regions where vegetation spans a grassland–forest gradient may be more complex in areas that have undergone recent woody encroachment [68]. Despite these challenges, several hotspots for restoration across our case study region are identified for each vegetation type. Some hotspots span regions of complex topography and high altitudes, such as the central hotspot in the Brazilian Highlands and eastern hotspot along the Serra do Espinhaço (electronic supplementary material, figure S12). Here, complex topography may have limited large-scale land-use change [69]. Hotspot areas also frequently coincide with legally protected areas where large expanses of native vegetation remain intact (electronic supplementary material, figure S2). When using this set of prioritization criteria, this suggests that the continued preservation and maintenance of protected areas are likely to be important for successfully achieving restoration targets across a range of vegetation types [70]. Transitional zones between different major biomes (the Cerrado, Mata Atlântica, Amazonia and the Caatinga in the northern, western and eastern hotspots) are also often identified as hotspot areas using this approach. In these transitional zones where disturbance of native savannahs and W&F is low, there may be potential to restore species associated with multiple biomes, also frequently coinciding with high biomass stocks [71,72]. The majority of the hotspot locations identified are, however, not in areas that are required to be restored under Brazil's Forest Code [43]. This suggests that current land use policies governing national restoration commitments, like the Forest Code, are not sufficient for optimizing restoration opportunities in some seasonally dry tropical biomes. Rather than focusing on areas that may optimize carbon and biodiversity gains and are easier to restore [2,38], they may allow the removal of native vegetation in these areas. This implies that current restoration policy within seasonally dry tropical regions may not always maximize restoration effectiveness across all vegetation types within the landscape. Furthermore, it suggests that achieving restoration in hotspot areas may presently be reliant on private stakeholders via schemes that offer payments for ecosystem services, further highlighting the need to engage a variety of groups to optimize returns from restoration in seasonally tropical dry regions [36].

The spatial distribution of hotspot areas is explained by the distribution of potential restoration benefits, which were considerable across our study region. Modelled local diversity was high for all three of the vegetation types (figure 2*a*–*c*). As expected, savannah vegetation had high local diversity due to the inclusion of both herbaceous and woody species. The modelled local diversity of W&F was also high due to the occurrence of multiple forest types within the region, dense savannah woodland areas and transitional areas between the Cerrado region and neighbouring biomes [11,73]. High altitude, mountainous and rocky outcrop hotspot areas (central and eastern hotspots) had a high modelled local diversity for grassland and savannah species (figure 2*b*,*c*, electronic supplementary material, figure S10). Local diversity and endemism are typically high in rock outcrop areas (see electronic supplementary material, table S1) [74]. However, relative to woody species, studies of the species richness and phytogeographic patterns of herbs and shrubs in the Cerrado are sparse, and in addition many species are rare, with narrow distributions, which makes representing them in SDMs more difficult [16,75]. This is likely to result in greater underestimation of the biodiversity benefits of restoring herb and shrub vegetation types. Furthermore, each area of high species richness falls within a different floristic biogeographic district [17] potentially maximizing β-diversity. The heterogeneous vegetation of the seasonally dry tropics, relative to moist tropical areas [31,32], means that restoration (and conservation) of a range of different vegetation types across the geographical extent of savannah-dominated regions is vital in the global effort to conserve and restore biodiversity [28,76].

In addition to their high species richness, the total biomass stocks of mature restored savannahs are considerable due to the high investment of savannah plants into belowground biomass (electronic supplementary material, table S2) [44]. Across regions with a high proportion of savannah cover, although challenging to measure directly, it will therefore be critical to accurately account for carbon sequestration into belowground biomass stocks when implementing restoration projects [77]. Although lower than the rainforests of Amazonia and Mata Atlântica [78], the potential total biomass stocks of restored W&F in the Cerrado are high, particularly at the transition zones at the edges of the region (electronic supplementary material, table S1 and figure S7) [44,72]. SOC content and soil quality indicators are often neglected in empirical tropical restoration studies [79] and understanding SOC gains or losses as a result of restoration will be critical for further prioritization attempts [80,81]. Although potential SOC gains from restoration are not included in our analysis, a recent study demonstrated that SOC gains in grassland vegetation under high CO_2_ could be greater than those of forested areas, suggesting that tropical grasslands may be more important for long-term C storage in soils [82]. Though frequently overlooked [9], appropriate restoration of all vegetation types within tropical savannah regions is important for achieving long-term emissions reduction targets.

Legal protection of remaining native vegetation in the Cerrado region is weak, especially when compared to neighbouring Amazonia ([43,83], electronic supplementary material, figure S10). Yet conservation of this vegetation, as is the case in other seasonally dry tropical regions, is essential to avoiding further biodiversity loss and increased CO_2_ emissions, alongside increasing the chances of restoration success through enhancing the potential for natural regeneration and reducing the probability of exotic pasture grass invasion [63,84]. Enhancing connectivity, which is more easily achieved in the northern Cerrado (figure 2*e*), facilitates migration and therefore gene flow, which is essential to building ecosystem resilience [76,85]. Conserved remaining native vegetation can also be a source of propagules for active restoration [28]. Fragmentation of remaining native vegetation is particularly high across the southern Cerrado, due to extensive urban, pastureland and agricultural expansion (figures 1*a* and 2*e*) [12]. Across highly degraded savannah-dominated regions, despite the greater availability of pastureland that could potentially be restored, the high proportion of exotic pasture grasses in the landscape matrix and the low availability of propagules will likely make restoration harder [27,29,63]. Greater landscape degradation will also necessitate more costly active restoration techniques making restoration more expensive [32,86].

We have demonstrated considerable potential benefits in carbon storage and plant species diversity through restoring a range of vegetation types in a seasonally dry savannah-dominated region and identified restoration hotspot areas. However, the specifics of how to define a hotspot are often dependent on the aims and requirements of the stakeholder implementing a restoration, as well as on data availability, and future prioritization attempts may be able to consider additional factors to maximize benefits. Potential gains in plant species richness have been considered here, but it is also reasonable to assume that ecosystem restoration will be beneficial for fauna as well [87]. While potential benefits from restoring native species and ecosystems across pasturelands have been quantified, additional benefits for landowners may be gained through the restoration of cropland areas with mixed planted systems including harvestable native species (ensuring the restoration vegetation type is appropriate) [88]. Future analysis should also consider the importance of native vegetation, particularly grasslands and savannahs, in providing and maintaining water security, especially in highly populated areas (e.g. the southern Cerrado) [45,89], and the potential for restoration to generate socioeconomic benefits [38]. Optimization of several benefits may be challenging outside of hotspot locations, as restoration benefits do not necessarily coincide across the majority of areas (electronic supplementary material, figure S9). Furthermore, given the estimated potential increases in ST_max_ by 2100 (under RCP 8.5, figure 5*b*), restoration in the Cerrado and other seasonally dry tropical regions [33] must be climate smart to ensure longevity. Across the seasonally dry tropics, the effects of rising temperatures and land management on fire frequency and water availability are likely to be complex [33,90], and assessing their effect on restoration projects and the potential of restoration to offset them is challenging and requires long-term ecological monitoring.

The potential benefits of heterogeneous landscape restoration across the geographical extent of seasonally dry tropical regions are clear, but restoration projects in these systems are rare relative to other regions. Evidence that the species composition and functioning of native reference systems can be restored through either active or passive restoration is lacking [24,25,29], and when considered alongside predicted climatic changes, the timescale at which these benefits might be realised is therefore challenging to assess.

## 5. Conclusion

Using Brazil's Cerrado region as an example, this study demonstrates that there is significant potential to initiate restoration projects across tropical savannah-dominated regions, which could generate long-term, national and international payback in ecosystem service generation and biodiversity conservation. Further, we highlight the importance of restoring all vegetation types across heterogeneous mosaic landscapes to maximize the paybacks from restoration. Four hotspot locations for restoration in the Cerrado region are identified where the gains in carbon sequestered in vegetation biomass and plant species richness could be maximized. These hotspots are in areas where the potential to connect restoration projects to remaining native vegetation are highest. Outside of these hotspots, active restoration techniques are likely to be required in the majority of areas as little native vegetation remains intact, emphasizing the importance of conserving remaining vegetation across highly degraded landscapes. Such multi-criteria hotspot locations may be ideal areas to focus initial restoration efforts, as we continue to work towards restoration targets.

## Data Availability

All datasets used and R codes used for the species distribution modelling can be accessed freely and are cited in the article or electronic supplementary material. The data are provided in the electronic supplementary material [91].
